# Characterization of the complete chloroplast genome of an endangered ornamental and medicinal plant *Lilium pumilum*

**DOI:** 10.1080/23802359.2020.1726228

**Published:** 2020-02-11

**Authors:** Jian Jin, Hao Liu, Can Zhong, Jing Xie, You Qin, Xuejuan Liang, Lin Chen, Ping Cai, Shuihan Zhang

**Affiliations:** a Institute of Chinese Materia Medica, Hunan Academy of Chinese Medicine, Changsha, PR China;; b Graduate School, Hunan University of Chinese Medicine, Changsha, P. R. China

**Keywords:** *Lilium pumilum*, High-throughput sequencing, Chloroplast, Genome sequence

## Abstract

*Lilium pumilum* DC. is a useful plant species not only for its showy flowers but also for its edible and medicinal values. Here we report on the complete chloroplast genome sequence of *L. pumilum*. The chloroplast genome is 152,573 bp in size and includes two inverted repeat regions of 52,984 bp, which is separated by a large single-copy region of 82,009 bp and a small single copy region of 17,580 bp. A total of 130 genes were predicted, including 38 tRNA, 8 rRNA, and 84 protein-coding genes. Phylogenetic analysis placed *L. pumilum* under the family Liliaceae.

*Lilium pumilum* DC. is a valuable plant species not only for its showy flowers but also for its edible and medicinal values (Tang et al. 2014). *L. pumilum* is striking due to its graceful flowers and bright red color. The bulbs *L. pumilum*, commonly known as “Bai-he” and recorded in Chinese Pharmacopeia, have been used as traditional Chinese medicines for the treating of various diseases long time (Ma et al. 2017). “Bai-he” is also regularly consumed as functional food and used to make Chinese cuisines and food supplements (Hwang et al. 2016; Lee et al. 2016; Song et al. 2016; Su et al. 2019).

Unfortunately, vegetative reproduction of *L. pumilum* is difficult and the populations of *L. pumilum* may have already disappeared due to overgrazing, uncontrolled overexploitation, and the effects of climate change (Zhang et al. 2016). Hence, it is necessary to develop the genetic resources to protect this endangered species. Here, we generated the complete chloroplast genome of *L. pumilum* for further research.

Total genomic DNA was extracted from fresh leaves of *L. pumilum* planted in Botanical Garden, Institute of Chinese Materia Medica, Hunan Academy of Chinese Medicine (N28°13′28.15″, E112°56′26.96″). Specimens were kept in in Hunan Herbarium of Chinese Traditional Medicine under the collection number HUTM100005.

TruSeq DNA Sample Prep Kit (Illumina, USA) was used to construct a genomic library consisting of an insert size of 350 bp. Sequencing was carried out on an Illumina NovaSeq platform. The output was a 6 Gb raw data of 150 bp paired-end reads, further trimmed and assembled using SPAdes (Bankevich et al. 2012). Annotations of chloroplast genome were conducted by the software Geneious (Kearse et al. 2012) and checked by comparison against the *Lilium brownii* complete chloroplast genome (GenBank accession number: KY748296).

The complete chloroplast genome of *L. pumilum* (GenBank accession number: MN906760) is 152,573 bp in length, displaying a quadripartite structure that contains a pair of inverted repeats (IR) regions (52,984 bp), separated by a large single-copy (LSC) region (82,009 bp) and a small single-copy (SSC) region (17,580bp). There are 130 genes reported, including 84 protein-coding genes, 8 ribosomal RNA genes, and 38 transfer RNA genes. The overall GC content of the chloroplast genome was 37.02%.

A maximum-likelihood tree was constructed with 1000 bootstrap replicates using FastTree software for phylogenetic analysis (Liu et al. 2011). A subset of 30 species from the family Liliaceae including 24 species from the genus *Lilium* was included, with species *polygonatum cyrtonema* from Asparagaceae as outgroup. The maximum-likelihood analysis showed that *L. pumilum* is placed under the family Liliaceae, clustered together with other *Lilium* species (Figure 1). The taxonomic status of *L. pumilum* exhibits a closest relationship with *L. amabile, L. callosum* and *L. lancifolium*. This finding could provide insight into conservation and exploitation efforts for this endangered ornamental and medicinal species.

**Figure 1.**
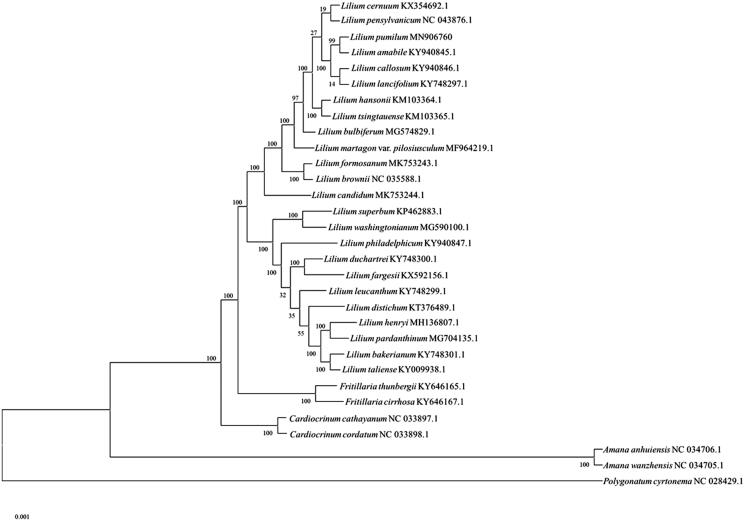
Maximum-likelihood tree based on the complete chloroplast genome sequences of 30 species from the family Liliaceae with *polygonatum cyrtonema* as outgroup. The bootstrap values were based on 1000 replicates.
